# A tool for self-assessment of communication skills and professionalism in residents

**DOI:** 10.1186/1472-6920-9-1

**Published:** 2009-01-08

**Authors:** Andrew B Symons, Andrew Swanson, Denise McGuigan, Susan Orrange, Elie A Akl

**Affiliations:** 1 Department of Family Medicine, University at Buffalo School of Medicine and Biomedical Sciences, Buffalo, New York, USA; 2 Graduate Medical Education, University at Buffalo School of Medicine and Biomedical Sciences, Buffalo, New York, USA; 3 Department of Medicine and Department of Family Medicine, University at Buffalo School of Medicine and Biomedical Sciences, Buffalo, New York, USA

## Abstract

**Background:**

Effective communication skills and professionalism are critical for physicians in order to provide optimum care and achieve better health outcomes. The aims of this study were to evaluate residents' self-assessment of their communication skills and professionalism in dealing with patients, and to evaluate the psychometric properties of a self-assessment questionnaire.

**Methods:**

A modified version of the American Board of Internal Medicine's (ABIM) Patient Assessment survey was completed by 130 residents in 23 surgical and non-surgical training programs affiliated with a single medical school. Descriptive, regression and factor analyses were performed. Internal consistency, inter-item gamma scores, and discriminative validity of the questionnaire were determined.

**Results:**

Factor analysis suggested two groups of items: one group relating to developing interpersonal relationships with patients and one group relating to conveying medical information to patients. Cronbach's alpha (0.86) indicated internal consistency. Males rated themselves higher than females in items related to explaining things to patients. When compared to graduates of U.S. medical schools, graduates of medical schools outside the U.S. rated themselves higher in items related to listening to the patient, yet lower in using understandable language. Surgical residents rated themselves higher than non-surgical residents in explaining options to patients.

**Conclusion:**

This appears to be an internally consistent and reliable tool for residents' self-assessment of communication skills and professionalism. Some demographic differences in self-perceived communication skills were noted.

## Background

Excellent communication skills are essential to the practice of medicine.[1] Effective communication during medical encounters has been associated with significant benefits in areas such as patient recall and understanding, adherence to treatment plans, symptom resolution, physiological outcomes, and medical decision-making, as well as satisfaction of both patients and physicians.[2,3]

Positive doctor-patient relations can increase the patient's perceptions of physician competence.[4] Research has shown that physicians who exhibit negative communication behaviors are more likely to have been sued in the past for malpractice than those with more positive doctor-patient relations. [5-8] Beckman, et al.[9] found that in 70% of malpractice depositions, communication problems between physicians and patients were identified.

Several national organizations have recognized the importance of fostering and evaluating communication skills and professionalism in physicians in training and in practice.[1,10-12] The Association of American Medical Colleges (AAMC) Medical School Objectives Project urges medical schools to teach these skills.[13] The Accreditation Council for Graduate Medical Education (ACGME) has defined these skills as core competencies that programs have to train in and evaluate.[14] Finally, the American Board of Internal Medicine (ABIM) includes an evaluation of communication skills and professionalism in the recertification process of practicing physicians.[15]

The wide agreement on the importance of communication skills and professionalism challenges medical educators to develop effective methods and tools to evaluate them. Current evaluation methods in residency training programs include attending physician assessment, peer evaluation, standardized patient assessment, actual patient assessment, and self-assessment. [16-18] One of the available evaluation tools is the ABIM Patient Assessment survey, a widely used instrument for patient evaluation of physicians. [19-23]

The aims of this study were to evaluate residents' self-assessment of their communication skills and professionalism in dealing with patients, and to evaluate the psychometric properties of the ABIM Patient Assessment survey, modified as a self-assessment questionnaire for this purpose. Self-assessment, though it has some limitations, [24-30] may be used as part of a multi-source evaluation scheme for those studying and practicing medicine.[1,10,31-35]

## Methods

### Setting and participants

The Office of Graduate Medical Education of the University at Buffalo School of Medicine and Biomedical Sciences organizes a yearly core curriculum "master session" on professionalism. Residents in all of the 23 affiliated training programs are required to attend this master session at least once by the time they graduate from their residency.

### Study design

We invited all residents attending the 2007 master session on professionalism to voluntarily participate in the study by completing a two-page anonymous questionnaire. We distributed the questionnaire at the beginning of the session along with other lecture-related material. At the end of the session, those who chose to participate left the survey in a box anonymously. The Institutional Review Board of the University at Buffalo approved the study.

### Survey questionnaire

The survey questionnaire was a modified version of the ABIM's Patient Assessment survey, which is part of the Patient and Physician Peer Assessment Module for maintenance of certification.[36] The Patient Assessment survey includes 11 questions about different aspects of communication skills and professionalism of the physician. Patients rate each of these aspects on five-point Likert scales ranging from 1 (poor) to 5 (excellent). Lipner[19] applied generalizability theory to the survey as administered to patients about their physicians. The variance component for participants was 0.01. The generalizability coefficient was 0.67 with a 95% confident interval of ± 0.14.

We modified the Patient Assessment survey to a physician self-assessment survey by using the third-person (e.g., "greeting them warmly") instead of the second-person (e.g., "greeting you warmly") while otherwise preserving the exact same wording. The questionnaire included additional questions related to resident demographic characteristics (age, sex), and educational characteristics (year of residency, country of graduation [U.S. versus international], number of years since medical school graduation, and specialty).

### Statistical analysis

We performed a descriptive analysis (frequencies and percentages) of the demographic and educational characteristics of the participants. We conducted factor analysis to search for items showing a high level of commonality. Prior to conducting the factor analysis, we tested the suitability of the scale using the Kaiser-Meyer-Oklin (KMO) test and the Bartlett's Test of Sphericity. Because the data were skewed toward the higher values, we re-categorized the answer choices into three groups: "poor or fair," "good," and "very good or excellent."

We then conducted a series of analyses to evaluate the psychometric properties of the survey questionnaire. We measured internal consistency reliability (Cronbach's alpha) to assess whether all the items were contributing to the measurement of professionalism. We also measured inter-item gamma scores to examine the level of relationship between the items in the questionnaire. Gamma replaced the standard Pearson *r *correlation due to the categorical nature of the results. We assessed association utilizing cross tabulation techniques with chi square statistics to determine the discriminative validity of the proposed instrument, regardless of the demographic and educational characteristics of the participants. In addition, we calculated the Kruskal-Wallis K Independent Samples test to examine differences in total score across demographic and educational groups. The total score was computed by tabulating the sum of all 11 items to provide a continuous measure of communications skills and professionalism. We used SPSS version 13.0 (SPSS, Inc., Chicago, Illinois) for all analyses.

## Results

Of 149 individuals who registered for the session, 132 participated in the survey but only 130 completed the entire questionnaire (87.2% response rate). Table 1 lists participants' demographic and educational characteristics.

**Table 1:** Demographic Characteristics

	**Frequency (N = 130)**	**Percent**
**Sex**		
Female	51	39.2
Male	79	60.8
**Post Graduation Year**		
Post-Year 1	30	23.1
Post-Year 2	42	32.3
Post-Year 3	30	23.1
Post-Year 4	14	10.8
Post-Greater than Year 4	14	10.8
**Medical Degree**		
Graduate of U.S. medical schools	61	46.9
Graduate of medical schools outside the U.S.	69	53.1
Type of Residency *		
Surgical or surgical subspecialty	44	34.1
Non-surgical specialty	85	65.9

* Missing value for n = 1

### Factor analysis

The scale was suitable for the factor analysis: the KMO test value was 0.806 (recommended minimum level is 0.6), and the Bartlett test value was statistically significant (p < 0.001).

Factor analysis revealed the presence of three components with eigen values over 1, explaining 60.5% of the total variance. These three components were groupings of questions based on common themes. Upon inspecting the scree plot obtained during initial analysis of the factors, a break was observed between factors two and three; thus it was decided to retain only the first two factors for investigation. The two retained factors accounted for a total of 51.4% of the variance. We defined these factors as items related to "Interpersonal Relations" and items related to "Conveying Medical Information." To further aid interpretation, Varimax rotation was performed. Table 2 shows the rotated factors, emphasizing a relatively simple structure with factors loading on only one component in all but two cases. Items loading strongly on component one (interpersonal relations) include Item 1 (being truthful, telling the patient everything, etc.), Item 2 (greeting them warmly, etc.), Item 3 (treating the patient on the same level, not "talking down", etc.), Item 4 (letting the patient tell his/her story, not interrupting, etc.), Item 5 (showing interest in the patient, not acting bored/uninterested), and Item 10 (using understandable words, explaining medical and technical terms).

**Table 2:** Rotated Component Matrix

	Interpersonal Relations 1	Conveying Medical Information 2
Item 2: Greeting them warmly; calling them by the name they prefer; being friendly, never crabby or rude	0.734	
Item 3: Treating them like they're on the same level; never "talking down" to them or treating them like a child	0.727	
Item 5: Showing interest in them as a person; not acting bored or ignoring what they have to say	0.659	
Item 1: Telling them everything; being truthful, upfront and frank; not keeping things from them that they should know	0.630	
Item 4: Letting them tell their story; listening carefully; asking thoughtful questions; not interrupting them while they're talking	0.613	
Item 10: Using words they can understand when explaining their problems and treatment; explaining any technical medical terms in plain language	0.563	0.369
Item 11: How would you rate your level of professionalism?	0.548	0.532
Item 9: Explaining what they need to know about their problems, how and why they occurred, and what to expect next		0.796
Item 7: Discussing options with them; asking their opinion; offering choices and letting them help decide what to do; asking what they think before telling them what to do		0.697
Item 6: Warning them during the physical exam about what you are going to do and why; telling them what you find		0.662
Item 8: Encouraging them to ask questions; answering them clearly; never avoiding their questions or lecturing them		0.659

Note: Only loadings above 0.3 are shown

Items loading strongly on component two (conveying medical information) include; Item 6 (warning the patient prior to a physical exam), Item 7 (discussing options with the patient, offering choices, etc.), Item 8 (encouraging the patient to ask questions), and Item 9 (explaining to the patient what they need to know about their medical issue). There was overlap on two items, Item 10 (using words the patient can understand) and Item 11 (overall level of professionalism). Of particular note is the overlap between the two components on Item 11 (overall level). Additional data should be collected to further examine this overlap.

Item 10 loads in both component 1 (interpersonal relations) and component 2 (conveying medical information) indicating that clarity in speaking is important in interpersonal relations and conveying medical information. However, it loads stronger in component 1 (0.563 versus 0.369).

### Psychometric properties of the survey questionnaire

The Cronbach's alpha level was 0.86, well above the minimally acceptable level of 0.7. In addition, each component's internal consistency reliability was assessed. Component 1 (Interpersonal Relations) showed an internal consistency reliability of 0.82 and component two (Communication and Professionalism) showed an internal consistency reliability of 0.80. Based on these results, both components show a high level of internal consistency reliability, suggesting that each component's items are measuring the main idea of the components.

The Kruskal-Wallis K Independent Samples test resulted in no significant differences between groups in each of the demographic and educational variables. The lack of significant differences shows that the total score of professionalism is not influenced in a significant way by membership in any one of these groups.

The gamma tests showed significant associations between some of the self-rating items and some of the educational and demographic characteristics. Males, compared with females, rated themselves significantly more positively on Item 1 (telling your patient everything, etc.; *p *= 0.032), Item 9 (explaining to your patient what they need to know, etc.; *p *= 0.040), and Item 10 (using words they can understand, etc; *p *= 0.017).

There were two statistically significant differences between graduates of U.S. medical schools and graduates of medical schools outside the U.S. International graduates, compared with U.S. graduates, rated themselves significantly more positively on Item 4 (letting patients tell their story, etc; *p *= 0.003). On the other hand, U.S. graduates, compared with international graduates, rated themselves significantly more positively on Item 10 (using words they can understand, etc.; *p *= 0.003).

Finally, residents in surgical specialties, compared with non-surgical specialties, rated themselves significantly more positively on Item 7 (discussing options with patients, etc.; *p *= 0.005).

## Discussion

The main utility of our educational exercise (the professionalism presentation) was to raise resident awareness regarding issues of doctor-patient communication and to expose them to a tool which has been used to survey patients about their perceptions of their doctors. Our psychometric analysis of the adaptation of the ABIM patient satisfaction survey for resident self-assessment shows highly acceptable levels of internal consistency reliability. In addition, factor analysis shows us the beginning development of two distinct components of professionalism ("interpersonal relations" and "conveying medical information"). These two components allow for the potential creation of separate sub-scales of professionalism. Of particular interest is the overlap on the two components of overall level of professionalism. This overlap could potentially be due to a number of influences. For instance, it could be that both subscales adequately measure overall professionalism and the item is important in both components. One might speculate that items related to "conveying information" are fairly mechanistic, whereas items related to "interpersonal relations" require being more in tune with the patient's emotions. Ginsburg and colleagues[37] noted that the nature of professionalism is context-specific. Perhaps physicians need to rely on a different set of communication skills depending on the context of the clinical interaction (e.g., delivering a poor prognosis for a cancer versus describing the risks/benefits/alternatives of epidural anesthesia during labor). The issue of "sub-scales" of professionalism could be explored further by administering the survey to patients and residents immediately after a clinical encounter and noting the context of the interaction.

The results also show significant associations between self ratings and certain participant characteristics. Males tended to rate themselves higher than females on certain items. While this might be their self-perception, reality may prove contrary. In a study by Minter,[38] female surgical residents were found to underestimate their skills compared to attending physician evaluation. Additional studies have found that females score better than males after a training course in communication skills, [39-42] female physicians engage in more patient-centered communication than do males,[43] and female graduates of medical schools outside the U.S. received slightly higher communication and interpersonal skills ratings than did male graduates of medical schools outside the U.S. on the United States Medical Licensing Examination™ Step 2 Clinical Skills exam.[44] When the original ABIM survey was administered to patients for purposes of assessing physicians, female doctors received higher overall professionalism ratings from their patients.[19]

Foreign graduates reported doing a better job at listening to their patients, whereas U.S. graduates reported using more understandable language. This may have more to do with confidence in English language proficiency rather than basic communication skills; foreign graduates might listen more closely so as not to misunderstand their patients, and their lack of confidence in speaking English might cause them to perceive that they are not speaking in language which is easily understandable to their patients. This would need to be pursued in a further study, particularly comparing resident self-assessment to actual patient perception.

Surgical residents rated themselves more positively in discussing options with patients compared to residents in non-surgical specialties. The quality of these communication skills has a substantial impact on patient outcomes, including superior recovery rates in patients undergoing surgical procedures,[45] reduced need for analgesic use to treat post-operative pain,[46] and improved emotional and functional adjustment in adult cancer patients.[47] We might speculate that surgery residents' perceptions of their ability to discuss options might be higher than non-surgical residents' because, often in surgery, the options are more concrete (i.e., surgery versus no surgery) whereas patient management options in the non-surgical specialties are often less concrete (i.e., one medication regime versus another medication regime) and perhaps more difficult to articulate to patients.

Current schemes for evaluating communication skills and professionalism in students and residents have flaws when used in isolation.[37] For instance, although standardized patients have been shown to be useful in evaluating the mechanics of clinical and communication skills,[48] their reliability in assessing elements of professionalism is less established. These settings may also be considered contrived. Faculty evaluations of professional behavior using instruments with Likert scales have been shown to have poor reliability, primarily because the attending physician usually does not interact extensively with the student in the work environment. [49-51] Peer evaluations, though they provide good information about interpersonal skills, are problematic because of the "halo effect" (i.e., if a person is popular, this "halo" may affect the evaluation of specific traits) and because peers are often reluctant to comment on poor behavior in colleagues.[1,10,16,52,53] Actual patient evaluations would seem to be the best source of evaluation of communication skills, but can be influenced greatly by the setting in which they are performed.

Recent reviews of self-assessment in the health professions raise questions about the ability of professionals to generate accurate judgments of their own performance.[24,25] Of even more concern is that those who perform the least well on external assessment may also overrate their performance on self-assessment exercises.[24,26-28] Most studies of self-assessment are in areas of technical knowledge and ability.[24] Even in concrete areas such as these, self-assessment has been found to be inaccurate.[29,30,37] This may be of even more concern when the area of assessment is laden with value judgments, as is the case in communication skills and professionalism.[54] In one study, medical residents perceived a high level of competence to discuss end-of-life issues, but failed to engage in recommended behaviors for such discussions.[55] When surveys use self-assessment, they are subject to social desirability bias.[1] This may limit the usefulness of these self-assessments to formative assessment and the formation of personal goals.[37]

It is thought that a more representative picture of professional behavior can be obtained by surveying a variety of patients and people in the healthcare team because they interact with students and residents on a more regular basis and may provide a more informative picture of professional behavior throughout their academic career.[1,10,31-33] This type of assessment is referred to as a "360-degree Evaluation." Resident self-assessment of professionalism skills may therefore be more useful when compared with the assessments made by others.[34,35]

There may also be educational benefit simply by the process of self-reflection. Reflection, both on the process and content of learning can help students to monitor their own learning.[56,57] Reflection-in-learning is related to readiness for self-regulation of learning and may be conducive to enhanced diagnostic ability.[58] Studies have found that a greater effort of reflection is associated with a more positive or meaningful learning experience.[58,59] The rationale for encouraging reflection in the promotion of self-directed learning is extensive.[60] Actually, reflection is conceived as one of the metacognitive skills or cognitive regulation strategies required for the development of self-regulated learning, from a theoretical viewpoint.[61]

## Conclusion

Through psychometric analysis, we have shown that the adaptation of the ABIM patient satisfaction survey is reliable for self-assessment of communication skills in residents. Although the face validity of the items has been reviewed elsewhere,[19] we hope to further establish the validity of this tool for resident self-assessment by administering the survey to patients and residents immediately following a clinical encounter, and comparing aggregate patient evaluations with the residents' own self-assessment of communication and professionalism skills. Although, as part of the ABIM Maintenance of Certification process, physicians are asked to answer the patient survey questions as a "self-assessment," we are aware of no published studies comparing the physician and patient data. We would like to explore comparing these data with results we have obtained or will obtain in our own studies of resident physicians. We would also like to use the resident self-assessment in conjunction with other resident evaluation methods currently in place at our institution.

## Competing interests

The authors declare that they have no competing interests.

## Authors' contributions

ABS: substantial contributions to research design, the acquisition, analysis and interpretation of data; drafting the paper; approval of the submitted and final versions. AS: substantial contributions to the interpretation of data; revising the paper critically; approval of the submitted and final versions. DM: substantial contributions to the interpretation of data; revising the paper critically; approval of the submitted and final versions. SO: acquisition of data, revising the paper critically; approval of the submitted and final versions. EAA: study design, data analysis and interpretation, revising the paper critically; approval of the submitted and final versions.

## Pre-publication history

The pre-publication history for this paper can be accessed here:


